# Surgery for Bismuth-Corlette Type 4 Perihilar Cholangiocarcinoma: Results from a Western Multicenter Collaborative Group

**DOI:** 10.1245/s10434-021-09905-z

**Published:** 2021-05-06

**Authors:** Andrea Ruzzenente, Fabio Bagante, Pim B. Olthof, Luca Aldrighetti, Ruslan Alikhanov, Matteo Cescon, Bas Groot Koerkamp, William R. Jarnagin, Silvio Nadalin, Johann Pratschke, Moritz Schmelzle, Ernesto Sparrelid, Hauke Lang, Calogero Iacono, Thomas M. van Gulik, Alfredo Guglielmi, Perihilar Cholangiocarcinoma Collaboration Group, A. Andreou, F. Bartsch, C. Benzing, S. Buettner, T. Campagnaro, I. Capobianco, R. Charco, P. de Reuver, E. de Savornin Lohman, C. H. C. Dejong, M. Efanov, J. I. Erdmann, L. C. Franken, G. Giovinazzo, M. C. Giglio, C. Gomez-Gavara, F. Heid, J. N. M. IJzermans, J. Isaac, H. Jansson, M. A. P. Ligthart, S. K. Maithel, M. Malagò, H. Z. Malik, P. Muiesan, S. W. M. Olde Damink, L. M. Quinn, F. Ratti, M. Ravaioli, J. Rolinger, E. Schadde, M. Serenari, R. Troisi, S. van Laarhoven, J. L. A. van Vugt

**Affiliations:** 1 Department of Surgery, Unit of Hepato-Pancreato-Biliary Surgery, University of Verona Medical School, Verona, Italy; 2 Department of Surgery, Erasmus Medical Center, Rotterdam, The Netherlands; 3 Department of Surgery, Amsterdam University Medical Center, University of Amsterdam, Amsterdam, The Netherlands; 4 Hepato-biliary Surgery Division, Ospedale San Raffaele-IRCCS, Milan, Italy; 5 Department of Hepato-Pancreato-Biliary Surgery, Moscow Clinical Scientific Center, Moscow, Russia; 6 Department of Medical and Surgical Sciences, S. Orsola-Malpighi Hospital, Alma Mater Studiorum – University of Bologna, Bologna, Italy; 7 Division of Hepatopancreatobiliary Surgery, Memorial Sloan-Kettering Cancer Center, New York, NY USA; 8 Department of General and Transplant Surgery, University Hospital Tübingen, Tübingen, Germany; 9 Department of Surgery, Charité - Universitätsmedizin Berlin, Berlin, Germany; 10 Department of Surgery, Centre for Digestive Diseases, Karolinska University Hospital, Stockholm, Sweden; 11 Department of General, Visceral and Transplantation Surgery, University Hospital of Mainz, Mainz, Germany

## Abstract

**Background:**

Although Bismuth-Corlette (BC) type 4 perihilar cholangiocarcinoma (pCCA) is no longer considered a contraindication for curative surgery, few data are available from Western series to indicate the outcomes for these patients. This study aimed to compare the short- and long-term outcomes for patients with BC type 4 versus BC types 2 and 3 pCCA undergoing surgical resection using a multi-institutional international database.

**Methods:**

Uni- and multivariable analyses of patients undergoing surgery at 20 Western centers for BC types 2 and 3 pCCA and BC type 4 pCCA.

**Results:**

Among 1138 pCCA patients included in the study, 826 (73%) had BC type 2 or 3 disease and 312 (27%) had type 4 disease. The two groups demonstrated significant differences in terms of clinicopathologic characteristics (i.e., portal vein embolization, extended hepatectomy, and positive margin). The incidence of severe complications was 46% for the BC types 2 and 3 patients and 51% for the BC type 4 patients (*p* = 0.1). Moreover, the 90-day mortality was 13% for the BC types 2 and 3 patients and 12% for the BC type 4 patients (*p* = 0.57). Lymph-node metastasis (N1; hazard-ratio [HR], 1.62), positive margins (R1; HR, 1.36), perineural invasion (HR, 1.53), and poor grade of differentiation (HR, 1.25) were predictors of survival (all *p* ≤0.004), but BC type was not associated with prognosis. Among the N0 and R0 patients, the 5-year overall survival was 43% for the patients with BC types 2 and 3 pCCA and 41% for those with BC type 4 pCCA (*p* = 0.60).

**Conclusions:**

In this analysis of a large Western multi-institutional cohort, resection was shown to be an acceptable curative treatment option for selected patients with BC type 4 pCCA although a more technically challenging surgical approach was required.

**Supplementary Information:**

The online version contains supplementary material available at 10.1245/s10434-021-09905-z.

Perihilar cholangiocarcinoma (pCCA) is a rare tumor originating from the epithelium of the hepatic duct confluence and commonly classified according to the Bismuth-Corlette classification (BC) based on the extent of proximal biliary infiltration.^1,2^ In particular, pCCA bilaterally involving the second-order biliary ducts has been classified as BC type 4. According to several prognostic staging systems (e.g., the American Joint Committee on Cancer [AJCC]–TNM 7th edition) as well as the American Hepato-Pancreato-Biliary Association (AHPBA) guidelines, BC type 4 has pCCA been considered a locally advanced tumor amenable to surgical treatments only for highly selected patients.^3,4^

Moreover, surgical resection of BC type 4 pCCA often requires extended hepatectomies (i.e., trisectionectomy), intrahepatic bile duct reconstruction, lymphadenectomy, and vascular resections that in addition to the innate technical challenges have been associated with a high risk of postoperative complications and mortality.^5,6^ Although BC type 4 therefore has represented a criterion for unresectability, recent experiences in Eastern and Western series have suggested that surgical resection with curative intent (i.e., negative margin [R0]) might offer a valid treatment option for this disease stage.^7–10^

Because recent improvements in surgical techniques and perioperative management of patients undergoing major liver surgery (i.e., extended hepatectomies with ≥5 liver segments) have reduced the rates of postoperative morbidity and mortality, several authors have currently supported resection with curative intent for patients with BC type 4 pCCA.^11^ In addition, instead of BC types, several variables, including surgical margin and lymph node status, have been associated with oncologic outcomes for pCCA patients undergoing surgery.^12,13^ For these reasons, in the new eighth edition of the AJCC TNM staging system, BC type 4 was removed as a criterion from the definition of stage T4 disease.^4^

To date, only few data have compared the short-term (i.e., morbidity and mortality) and long-term (i.e., overall and disease-free survival) outcomes of liver surgery with radical intent for BC type 4 tumors in the Western series. For these reasons, the current study, using a multi-institutional international database, aimed to assess the overall complications and mortality of BC type 4 pCCA treated with curative-intent surgical resection compared with those of BC types 2 and 3 pCCA. In addition, oncologic outcomes, including overall and disease-free survival, were compared.

## Patients and Methods

Patients who underwent surgical resection at one of the 20 tertiary referral hepato-biliary Western centers were included in the study (Supplementary Material). Participating centers had a median of 80 (range 25–115) consecutive resections for pCCA between January 2000 to December 2017. The study defined pCCA as a biliary tumor involving the hepatic duct confluence according to the definition of the Japanese Society of Biliary Surgery (JSBS).^14^ The study excluded all patients who underwent explorative surgery only, resection with macroscopic residual tumor (R2), or excision of only the extra-hepatic bile ducts or transplantation, as well as patients with metastases or distant lymph-node metastases (N2). Furthermore, patients with BC type 1 pCCA were excluded from the study given the relative heterogeneity in the surgical approach for this type of pCCA (i.e., bile duct resection only). The institutional review board of the participating institutions approved the study.

The multicenter design of the current study clearly implied differences in the preoperative management of the included patients. The type of preoperative evaluation of liver function varied among the different centers according to surgeon and center preferences, whereas future remnant liver volume (FRLV) was evaluated with computed tomography (CT) volumetry. Therefore, the selection of patients for preoperative portal vein embolization (PVE) and biliary drainage differed between centers according to center and surgeon preferences. In general, most patients for whom major liver resections were planned underwent preoperative, endoscopic, or transhepatic biliary drainage of at least the future remnant liver.

Preoperative cholangitis was defined as fever and leukocytosis requiring (additional) biliary drainage in accordance with the definitions applied in the DROP and DRAINAGE trials dealing with preoperative biliary drainage.^15,16^ Bismuth-Corlette (BC) classification was determined using preoperative imaging studies including contrast-enhanced CT, magnetic resonance imaging (MRI), MR cholangiography, and when available, endoscopic retrograde cholangiopancreatography (ERCP). Bismuth-Corlette type 4 pCCA was defined as tumor infiltration extending proximally into the segmental biliary ducts on both sides of the liver, usually including segment 1. The BC classification was locally assessed and then surgically confirmed after the surgical operation. Type of surgery, histologic data, and postoperative outcomes were recorded and analyzed in the collective database. Major and extended liver resections were defined as resections of at least three and five Couinaud liver segments, respectively. In the respective pathology reports, R0 resection was defined as tumor-free margins in all the reported margins (biliary and circumferential margins).

All complications after surgery were collected and classified according to the Clavien-Dindo classification system, with grade 3 or higher considered as major morbidity.^17^ Liver failure, biliary leakage, and hemorrhage were scored and classified according to the respective International Study Group of Liver Surgery (ISGLS) criteria, and only grades B and C were considered clinically relevant.^18–20^ Perioperative mortality was defined as death within 90 days after surgery.

### Statistical Analyses

Continuous variables were reported as medians with interquartile ranges (IQRs), whereas categorical variables were reported as totals and frequencies. Comparisons between categorical variables were assessed using the chi-square test or Fisher’s exact test, as appropriate. The outcomes for the survival analysis were overall survival (OS) and disease-free survival (DFS). The study defined OS as the interval between the date of surgery and the date of the patient’s death and recurrence as the interval between the date of surgery and the date or recurrence.. At the date of the last follow-up visit, OS was censored for the patients who were alive, and DFS was censored for those who remained disease-free. Both OS and DFS were estimated by Kaplan-Meier methodology, and survival curves were compared using log-rank analysis.

Cox proportional hazards regression analysis was used to evaluate any association among variables and survival outcomes, with coefficients reported as hazard ratios (HRs) and corresponding 95% confidence intervals (CIs). Variables with a *p* value lower than 0.1 in univariable analysis were included in the final multivariable models. Akaike information criterion (AIC) model selection was used to distinguish among a set of possible models describing the relationship between OS (and DFS) and the variables with *p* value lower than 0.1 in the univariable analysis. The best-fit model carrying the lowest AIC score was selected as the final model.

To account for potential residual confounders regarding the effect of surgical approach on outcomes, propensity scores were estimated using a logistic regression model with BC type as a dependent variable specified as type 4 versus types 2 and 3 pCCA. Gender, age, American Society of Anesthesiology (ASA) score, Ca 19-9 serum level, drainage, preoperative cholangitis, and PVE were independent variables in the logistic regression model. An exact propensity score value was used for matching. The degrees of covariate imbalance were measured using the standardized (mean and proportion) differences, as proposed by Austin^21^ A *p* value lower than 0.05 (two-tailed) was considered statistically significant. All analyses were performed using STATA version 12.0 (StataCorp LP, College Station, TX, USA) and R version 3.6.1 (2019-07-05) “Action of the Toes,” with the additional packages (i.e., survival and matching packages).^22^

## Results

### Patients’ Baseline Characteristics of BC Type 4 pCCA

Among the 1138 patients who underwent surgery for pCCA, 312 (27.4%) had BC type 4 tumors, whereas 826 (72.6%) had BC type 2 or 3 tumors (Table 1). The majority of the BC type 4 pCCA patients were male (*n* = 179, 57.4%), and about half of the patients were older than 65 years (*n* = 155, 49.7%). Of the 284 BC type 4 patients (91%) who required preoperative biliary drainage, 20.8% (*n* = 65) were treated with percutaneous transhepatic biliary drainage (PTBD) and 48.7% (*n* = 152) were treated with endoscopic biliary drainage. The vast majority of the patients (97.1%; *n* = 303) underwent major hepatectomy (≥3 liver segments), whereas 190 of the patients (60.9%) underwent an extended liver resection (≥5 liver segments).

**Table 1:** Baseline characteristics of perihilar cholangiocarcinoma patients with Bismuth type 4 (*n* = 312) versus Bismuth type 2 or 3 (*n* = 826) disease

Variables	Bismuth type 4	Bismuth type 2 or 3	*p* value
	*n* (%)	*n* (%)	
No. of patients	312	826	–
Age (years)			0.43
≤65	157 (50.3)	394 (47.7)	
>65	155 (49.7)	432 (52.3)	
Gender			0.94
Male	179 (57.4)	472 (57.1)	
Female	133 (42.6)	354 (42.9)	
ASA physical status classification			0.99
1–2	189 (62.4)	475 (62.3)	
3–4	114 (37.6)	287 (37.7)	
Preoperative biliary drainage			0.001
No	28 (9.0)	127 (15.4)	
Yes	284 (91.0)	699 (84.6)	
Type of biliary drainage			< 0.001
No drainage	28 (9.0)	127 (15.40)	
PTBD	65 (20.8)	238 (28.9)	
Endoscopic biliary drainage	152 (48.7)	339 (41.2)	
Both	67 (21.5)	119 (14.5)	
Preoperative cholangitis			0.95
No	237 (77.5)	600 (77.8)	
Yes	69 (22.5)	171 (22.2)	
Portal vein embolization			< 0.001
No	230 (73.7)	681 (82.4)	
Yes	82 (26.3)	145 (17.6)	
CA 19-9 ( U/mL)			0.17
≤100	216 (69.2)	536 (64.9)	
>100	96 (30.8)	290 (35.1)	
Major liver resection (≥ 3 segments)			0.2
Yes	303 (97.1)	812 (98.3)	
No	9 (2.9)	14 (1.7)	
Extended liver resection (≥ 5 segments)			< 0.001
Yes	190 (60.9)	383 (46.4)	
No	122 (39.1)	443 (53.6)	
Type of liver resection			< 0.001
Left hepatectomy	66 (21.1)	286 (34.6)	
Extended left hepatectomy	81 (26.0)	121 (14.6)	
Right hepatectomy	47 (15.1)	143 (17.3)	
Extended right hepatectomy	109 (34.9)	262 (31.8)	
Segments 4 and 5	3 (1.0)	9 (1.1)	
Central hepatectomy	6 (1.9)	5 (0.6)	
Caudate lobe resection			0.25
Yes	141 (45.2)	491 (59.4)	
No	52 (16.7)	146 (17.7)	
NA	119 (38.1)	189 (22.9)	
Portal vein resection			< 0.001
Yes	155 (49.7)	244 (29.5)	
No	157 (50.3)	582 (70.5)	
Hepatic artery resection			0.16
Yes	13 (4.2)	21 (2.5)	
No	299 (95.8)	805 (97.5)	
Perineural invasion			0.43
Yes	204 (65.4)	532 (64.4)	
No	57 (18.3)	176 (21.3)	
NA	51 (16.3)	118 (14.3)	
Tumor grade			0.61
Well/moderately	222 (71.2)	610 (73.8)	
Poor/undifferentiated	70 (22.4)	172 (20.8)	
NA	20 (6.4)	44 (5.4)	
Surgical margins			0.014
R0	190 (60.9)	567 (68.6)	
R1	122 (39.1)	259 (31.4)	
Lymphnodes status			0.23
N0	170 (54.5)	483 (58.5)	
N1	142 (45.4)	343 (41.5)	
Median hospital stay: days (IQR)	14.5 (10.0–24.0)	12.0 (11.0–15.0)	< 0.001
Post-hepatectomy liver failure			0.25
No failure/PHLF A	251 (80.4)	687 (83.4)	
PHLF B/C	61 (19.6)	137 (16.6)	
Biliary leak			0.1
No leak/grade A	240 (76.9)	669 (81.3)	
Grade B/C	72 (23.1)	154 (18.7)	
Intraabdominal abscess^a^			0.16
No	291 (93.3)	749 (90.7)	
Yes	21 (6.7)	77 (9.3)	
Severe bleeding complications^a^			0.3
No	301 (96.5)	785 (95.0)	
Yes	11 (3.5)	41 (5.0)	
Other severe complications^a^			0.3
No	264 (84.6)	713 (86.3)	
Yes	48 (15.4)	113 (13.7)	
Clavien-Dindo ≥3 complications			0.099
No	152 (48.7)	446 (54.2)	
Yes	160 (51.3)	377 (45.8)	
No. of severe complications^a^			0.3
1	131 (81.9)	283 (75.5)	
2	24 (15.0)	76 (50.5)	
3	5 (3.1)	12 (3.2)	
4	-	3 (0.8)	
30-Day mortality			0.36
No	284 (91.0)	767 (92.9)	
Yes	28 (9.0)	59 (7.1)	
90-Day mortality			0.57
No	274 (87.8)	715 (86.6)	
Yes	38 (12.2)	111 (13.4)	

ASA, American Society of Anesthesiologists; PTBD, percutaneous transhepatic biliary drainage; NA, not available; IQR, interquartile range; PHLF, post-hepatectomy liver failure

^a^Clavien-Dindo ≥3 complications

The analysis included 9 rare cases of patients (2.9%) who underwent central hepatectomy (<3 segments) and 113 cases of patients (36.2%) who underwent left and right hemi-hepatectomy (<5 segments). Interestingly, the incidence of positive margin (R1) did not differ between the two groups (R1: 2 [22.2%] without major hepatectomy vs 120 [39.6%] with major hepatectomy; *p* = 0.29; R1: 54 [44.3%] without extended hepatectomy vs 68 [35.8%] with extended hepatectomy; *p* = 0.13).////

Vascular resection/reconstruction was required for 163 patients (52.2%) including 150 cases of portal vein resection/reconstruction only (48.1%), 8 cases of hepatic artery resection/reconstruction only (2.5%), and 5 cases of combined portal vein and hepatic artery resection/reconstruction (1.6%). The overall incidence of severe complications (Clavien-Dindo ≥3) was 51.3% (*n* = 160), and the overall incidence of 90-day mortality was 12.2% (*n* = 38).

Among 61 (19.6%) patients who had a PHLF, 11 (18.0%, 11/61) had an associated intra-abdominal abscess. Moreover, 198 patients (17.4%) had a median hospital stay (LoS) of 14.5 days (IQR, 10–24 days), with 71.4% (*n* = 223) having an LoS longer than 10 days. During a median follow-up period of 21 months, the median overall survival (OS) was 28.4 months (95% CI, 23.7–33.0 months), and the median disease-free survival (DFS) was 26.3 months (95% CI, 19.0–33.7 months).

### Comparison of BC Type 4 With BC Types 2 and 3 pCCA

The patients with BC type 4 and those with BC type 2 or 3 pCCA demonstrated differences in terms of baseline clinical and pathologic characteristics (Table 1). In particular, the two groups, differed significantly in the use of PVE (BC type 4: *n* = 82 [26.3%]; BC type 2 or 3: *n* = 145 [17.6%]; *p* < 0.001), extended liver resection (BC type 4: *n* = 190 [60.9%]; BC type 2 or 3, *n* = 383 [46.4%]; *p* < 0.001), and positive margin status (R1; BC type 4, *n* = 122 [39.1%]; BC type 2 or 3, *n* = 259 [31.4%]; *p* = 0.014). Conversely, the BC type 4 patients and the BC types 2 and 3 patients had comparable incidences of severe complications (Clavien-Dindo ≥3) (BC type 4, *n* = 160 [51.3%]; BC types 2 and 3, *n* = 377 [45.8%]; *p* = 0.099) and 90-day mortality (BC type 4, *n* = 38 [12.2%]; BC types 2 and 3, *n* = 111 [13.4%]; *p* = 0.574) (Table 1). In particular, the BC type 4 patients and the BC types 2 and 3 patients had similar incidences of PHLF, biliary leak, intra-abdominal abscess, severe bleeding, other severe complications, and number of severe complications (all *p* > 0.1; Table 1).

### Overall Survival Analysis of the pCCA Patients

**Fig. 1:**
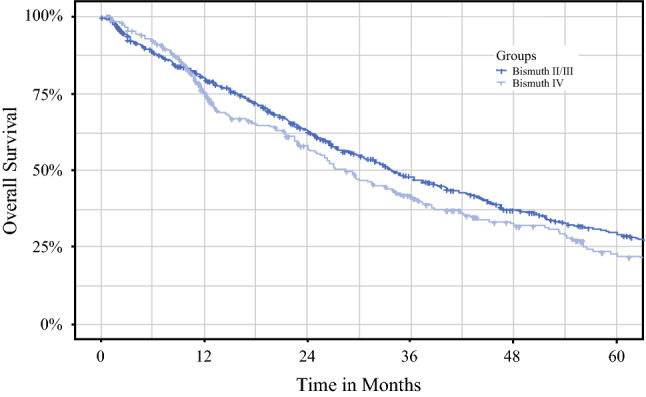
Overall survival of Bismuth type 2 or 3 patients versus Bismuth type 4 patients.

To investigate the oncologic outcomes for the BC type 4 patients and the BC types 2 and 3 patients, uni- and multivariable survival analyses were performed. The two groups had comparable OS (*p* = 0.063) and DFS (*p* = 0.94), with a median OS of 28.4 months (95% CI, 23.7–33.0 months) for the BC type 4 patients and 33.8 months (95% CI, 30.0–37.7 months) for the BC types 2 and 3 patients (Fig. 1). Similarly, the median DFS was 26.3 months (95% CI, 19.0–33.7 months) for the BC type 4 patients and 29 months (95% CI, 24.2–31.0 months) for the BC types 2 and 3 patients.

In the univariable survival analysis, several factors were associated with OS including PVE (HR, 1.21; 95% CI, 1.00–1.47; *p* = 0.050), portal vein (HR, 1.19; 95% CI, 1.02–1.40; *p* = 0.028), hepatic artery resections (HR, 1.62; 95% CI, 1.06–2.49; *p* = 0.025), positive margins (HR, 1.64; 95% CI, 1.39–1.93, *p* < 0.001), involvement of regional lymph nodes (HR, 1.87; 95% CI, 1.59–2.19, *p* < 0.001), presence of perineural invasion (HR, 1.66; 95% CI, 1.32–2.08; *p* < 0.001), and poor differentiation grade (HR, 1.39; 95% CI, 1.22–1.59; *p* < 0.001). Interestingly, OS was not associated with major liver resections (HR, 0.79; 95% CI, 0.45**–**1.37; *p* = 0.41) or extended liver resections (HR, 0.82; 95% CI, 0.47–1.43; *p* = 0.50) (Table 2).

**Table 2:** Uni- and multivariable survival analysis: Cox’s proportional hazard model for overall survival

Variables	Univariable		Multivariable	
	HR (95% CI)	*p* value	HR (95% CI)	*p* value
Gender (male vs female)	1.08 (0.94–1.26)	0.33		
Bismuth type (2 or 3 vs 4)	0.85 (0.71–1.00)	0.063		
Biliary drainage (yes vs no)	1.08 (0.95–1.13)	0.41		
Portal vein embolization (yes vs no)	1.21 (1.00–1.47)	0.050		
Major liver resections (yes vs no)	0.79 (0.45–1.37)	0.41		
Extended liver resections (yes vs no)	0.82 (0.47–1.43)	0.50		
Segment 1 resected (yes vs no)	0.97 (0.79–1.20)	0.81		
Portal vein resection (yes vs no)	1.19 (1.02–1.40)	0.028		
Hepatic artery resection (yes vs no)	1.62 (1.06–2.49)	0.025		
Margins (positive vs negative)	1.64 (1.39–1.93)	<0.001	1.36 (1.10–1.67)	0.004
N stage (N1 vs N0)	1.87 (1.59–2.19)	<0.001	1.62 (1.32–1.99)	<0.001
Perineural invasion (yes vs no)	1.66 (1.32–2.08)	<0.001	1.53 (1.15–2.04)	0.004
Differentiation grade (poor vs well/moderately)	1.39 (1.22–1.59)	<0.001	1.25 (1.05–1.47)	0.009

HR, hazard ratio; CI, confidence interval

The Akaike information criterion (AIC) model selection was used to identify the best-fit model**.** In the multivariable analysis, OS was associated with positive margins (R1: HR, 1.36; 95% CI, 1.10–1.67; *p* = 0.004), lymph node metastasis (N1: HR, 1.62; 95% CI, 1.32–1.99; *p* < 0.001), presence of perineural invasion (HR, 1.53; 95% CI, 1.15–2.04; *p* = 0.004), and poorly differentiated tumor grade (HR, 1.25; 95% CI, 1.05–1.47; *p* = 0.009). Notably, BC type 4 pCCA was not associated with a poorer prognosis than BC types 2 and 3 pCCA (*p* = 0.063).

### Disease-Free Survival Analysis of the pCCA Patients

In the univariable survival analysis, several factors were associated with DFS including male gender (HR, 1.30; 95% CI, 1.05–1.61; *p* = 0.015), positive margins (R1: HR, 1.63; 95% CI, 1.30–2.03; *p* < 0.001), lymph node metastasis (N1: HR, 1.86; 95% CI, 1.51–2.30; *p* < 0.001), extended liver resection (HR, 0.72; 95% CI, 0.59–0.89; *p* = 0.003), and poorly differentiated tumor grade (HR, 1.40; 95% CI, 1.16–1.69; *p* < 0.001). Interestingly, major hepatectomy was not associated with DFS (HR, 1.65; 95% CI, 0.61–4.35; *p* = 0.33; Table 3).

**Table 3:** Uni- and multivariable survival analysis: Cox’s proportional hazard model for disease-free survival

Variables	Univariable		Multivariable	
	HR (95% CI)	*p* value	HR (95% CI)	*p* value
Gender (male vs female)	1.30 (1.05–1.61)	0.015	1.34 (1.03–1.75)	0.027
Bismuth type (2 or 3 vs 4)	1.00 (0.80–1.26)	0.94		
Biliary drainage (yes vs no)	0.94 (0.83–1.07)	0.41		
Portal vein embolization (yes vs no)	0.87 (0.67–1.14)	0.34		
Major liver resections (yes vs no)	1.65 (0.61–4.35)	0.33		
Extended liver resections (yes vs no)	0.72 (0.59–0.89)	0.003	0.73 (0.56–0.95)	0.020
Segment 1 resected (yes vs no)	1.07 (0.83–1.39)	0.57		
Portal vein resection (yes vs no)	0.82 (0.65–1.02)	0.078		
Hepatic artery resection (yes vs no)	1.11 (0.57–2.17)	0.74		
Margins (positive vs negative)	1.63 (1.30–2.03)	<0.001	1.46 (1.12–1.90)	0.005
N stage (N1 vs N0)	1.86 (1.51–2.30)	<0.001	1.73 (1.34–2.25)	< 0.001
Perineural invasion (yes vs no)	1.32 (0.98–1.78)	0.059		
Differentiation grade (poor vs well/moderately)	1.40 (1.16–1.69)	<0.001	1.52 (1.21–1.90)	< 0.001

HR, hazard ratio; CI, confidence interval

In the multivariable Cox regression analysis, DFS was associated with male gender (HR, 1.34; 95% CI, 1.03–1.75; *p* = 0.027), positive margins (R1: HR, 1.46; 95% CI, 1.12–1.90; *p* = 0.005), lymph node metastasis (N1: HR, 1.73; 95% CI, 1.34–2.25; *p* < 0.001), extended liver resection (HR, 0.73; 95% CI, 0.56–0.95; *p* = 0.020), and poorly differentiated tumor grade (HR, 1.52; 95% CI, 1.21–1.90; *p* < 0.001). Notably, BC type 4 pCCA was not associated with a higher risk of recurrence than BC types 2 and 3 pCCA (HR, 1.00; 95% CI, 0.80–1.26; *p* = 0.936; Table 3). These results were confirmed in the survival analysis for OS and DFS after a propensity score-matching (PSM) analysis among the two groups (Tables S1, S2, S3; Fig. S1). In particular, a 1:1 exact PSM was performed, identifying 306 BC type 4 patients and 306 BC types 2 and 3 patients, with no differences in terms of preoperative clinicopathologic characteristics (all *p* ≥ 0.99; Table S1). After PSM, the median LoS was 16 days (IQR, 10.0–26.0 days) for the BC type 4 patients compared with 12 days (IQR, 11.0–13.0 days) for the BC types 2 and 3 patients (*p* < 0.001). Moreover, compared with the BC types 2 and 3 patients, the BC type 4 patients had greater incidences of PHLF (PHLF B/C: BC type 4, *n* = 60 [19.6%]; BC types 2 and 3, *n* = 36 [11.8%]; *p* = 0.008), biliary leak (biliary leak B/C: BC type 4, *n* = 71 [23.2%]; BC types 2 and 3, *n* = 49 [16.1%]; *p* = 0.027), and severe complications (Clavien-Dindo ≥3: BC type 4, *n* = 157 [51.3%]; BC types 2 and 3, *n* = 122 {40%]; *p* = 0.005). Conversely the two groups had similar incidences of 30- and 90-day mortality (all *p* > 0.13). Moreover, BC type was not associated with OS or DFS in the uni- and multivariate analyses (all *p* > 0.38; Tables S2 and S3).

### Prognosis of the N0 BC Type 4 and BC Types 2 and 3 Patients

**Fig. 2:**
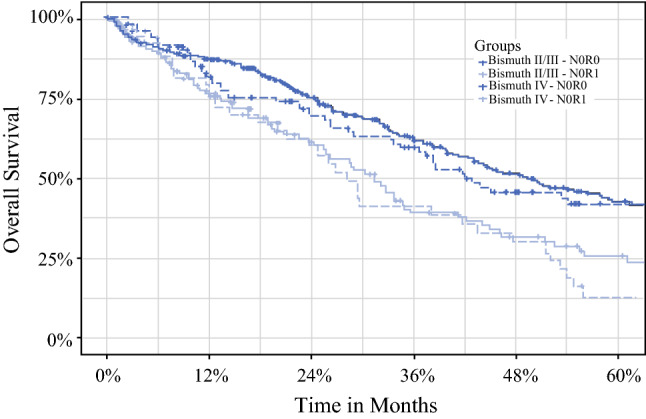
Overall survival of Bismuth type 2 or 3 patients versus Bismuth type 4 N0R0 patients.

For further investigation of the survival outcomes for the BC type 4 patients and BC types 2 and 3 patients, a sub-analysis including only patients with no lymph node metastasis (N0) was performed. Among the N0 patients with negative surgical margins (R0), the 5-year OS rate was 43% (95% CI, 36.7–50.3%) for the BC types 2 and 3 pCCA patients and 42.3% (95% CI, 32.5–55.0%) for the BC type 4 pCCA patients (*p* = 0.38). Among N0 patients with positive surgical margins (R1), the 5-year OS rate was 26% (95% CI, 18.0–37.5) for the BC types 2 and 3 pCCA patients and 13.4% (95% CI, 5.8–31.0) for the BC type 4 pCCA patients (*p* = 0.40; Fig. 2 and Fig S1).

In the Cox survival model, compared with N0/R0 BC types 2 and 3 patients, the N0/R1 BC types 2 and 3 pCCA patients and the BC type 4 pCCA patients had a 61% and 93% greater risk of death, respectively (N0/R1: BC types 2 and 3 pCCA: HR, 1.61; 95% CI, 1.22–2.11; *p* < 0.001; N0/R1 BC type 4 pCCA: HR, 1.93; 95% CI, 1.35–2.77; *p* < 0.001), whereas the N0/R0 BC type 4 patients had a similar prognosis (N0/R0 BC type 4 pCCA: HR, 1.14; 95% CI, 0.85–1.54; *p* = 0.39).

## Discussion

Although the use of extended liver resections for pCCA has continued to increase worldwide during the last decade, BC type 4 pCCA generally has been considered a locally advanced tumor amenable to surgical treatments only for highly selected patients treated by surgeons with challenging technical skills.^11,23^ Although pCCA often requires extensive surgery and a complex preoperative workup, patients with BC type 4 pCCA might require even a higher rate of extended hepatectomies to achieve complete resection (R0).^24,25^ Reportedly, additional vascular resections, including portal vein and hepatic artery resection/reconstruction, frequently achieve negative surgical margins (R0), increasing the complexity of surgery and resulting in higher incidences of complications and postoperative mortality.^24–26^ Recently, surgical series from Asian centers have shown that good surgical outcomes can be achieved also for patients with BC type 4 pCCA, providing evidence that surgical resection might offer a chance for long-term survival also for these patients with advanced tumors.^6,11^

In the current study, including 1138 patients who underwent surgical resection for pCCA at 20 Western centers, surgery for BC type 4 pCCA was associated with short-term (i.e., morbidity and mortality) and long-term (i.e., OS and DFS) outcomes comparable with those achieved by surgery for BC types 2 and 3 pCCA patients. Given that the differences in baseline characteristics (e.g., rate of extended hepatectomies) between the two groups might have influenced the analysis of the short-term outcomes, especially the long-term oncologic outcomes after surgery, a 1:1 PSM was performed to mitigate measurable baseline confounding in the comparison of the BC type 4 group and the BC types 2 and 3 group. After PSM, short-term (i.e., morbidity and mortality) and long-term (i.e., OS and DFS) outcomes were comparable between the BC type 4 pCCA patients and the BC types 2 and 3 pCCA patients.

In our analysis, the patients with BC type 4 pCCA and those with BC types 2 and 3 pCCA demonstrated differences in terms of clinical and pathologic characteristics including incidences of PVE (BC type 4 [26%] vs BC types 2 and 3 [18%]; *p* < 0.001), extended liver resection (BC type 4 [61%] vs BC types 2 and 3 [46%]; *p* < 0.001), and positive surgical margin (R1: BC type 4 [39%] vs BC types 2 and 3 [31%]; *p* = 0.014).

In comparison, Ebata et al.^27^ investigating the surgical outcomes for 1399 pCCA patients who underwent surgical resection at eight South-East Asian institutions reported that BC type 4 pCCA was associated with a higher incidence of lymph node metastasis (pN1: BC type 4 [54%] vs BC type 3 [39%]; *p* < 0.001) and distant metastasis (pM1: BC type 4 [16%] vs BC type 3 [9%]; *p* < 0,001) as well as a higher incidence of positive surgical margins (R1/R2: BC type 4 [37%] vs BC type 3 [20%]; *p* < 0.001).

Interestingly, in a recent review by Ku et al.^17^ including 292 BC type 4 pCCA patients who underwent liver surgery, the incidence of negative surgical margins (R0) ranged from 51% to 100%. When only the larger case series with more than 100 patients per center were considered, the incidence of R0 resections ranged from 63% to 75% and was comparable with that of our series (R0: BC type 4 [63%]).^27–29^ Conversely, in our analysis, the incidence of lymph node metastases (N1) was comparable between the BC type 4 and BC types 2 and 3 pCCA patients (N1: BC type 4 [45%] vs BC types 2 and 3 [41%]; *p* = 0.23), probably because of more strict selection criteria adopted in the Western centers for patients with BC type 4 tumors considered for resection.

In our series, the incidence of severe complications (Clavien-Dindo ≥3) was lower but not significantly different statistically for the BC types 2 and 3 patients than for the BC type 4 patients (complications: BC types 2 and 3 [46%] vs BC type 4 [51%]; *p* = 0.099), whereas the 90-day mortality did not differ between the two groups (90-day mortality: BC type 4 [12%] vs BC types 2 and 3 [13%]; *p* = 0.57). A 1:1 PSM analysis showed that the incidence of severe complications (Clavien-Dindo ≥3) and 90-day mortality also were comparable between the BC type 4 and BC types 2 and 3 patients (BC type 4 [48%] vs BC types 2 and 3 [46%], *p* = 0.82; 90-day mortality: BC type 4 [11%] vs BC types 2 and 3 [11%]; *p* > 0.99).

In a recent systematic review and meta-analysis of morbidity and mortality after major liver resection for pCCA patients, Franken et al.^30^ investigated the surgical outcomes for 4634 patients. Interestingly, whereas the pooled, overall incidence of severe morbidity (defined as Clavien-Dindo grade ≥3 or major complications requiring surgical, endoscopic, or radiologic reintervention, and/or life-threatening complications) was 40% (95% CI, 34–47%) in the sub-analysis including only the Western series, the incidence of severe morbidity was 43% (95% CI, 34–52%), similar to our results (Clavien-Dindo ≥3 complications: BC type 4: pre-PSM [51%]; post-PSM [48]%).^30^ In the same review, the pooled 90-day mortality was 9% (95% CI, 6–12%) among the Western centers, comparable with the incidence of 90-day mortality (12%; 95% CI, 10–15%) in our study (90-day mortality: BC type 4 patients: pre-PSM [12%]; post-PSM [11%]).^30^

Olthof et al.,^31^ investigating the role of PVE in patients undergoing surgery for pCCA, found that PVE before major liver resection was associated with a lower incidence of liver failure (PVE, 8% vs no PVE, 36%; *p* < 0.001) and mortality (PVE, 7% vs no PVE, 18%; *p* = 0.03). These results confirm a significant difference in morbidity and mortality compared with the postoperative outcomes reported by Western and Eastern centers.

The current study confirmed that curative surgery for pCCA, even in specialized tertiary Western centers, still is associated with high complication rates. Differences in patient characteristics (i.e., incidence of obesity) and preoperative treatment approaches (i.e., incidence of preoperative biliary drainage, type of biliary drainage used, and PVE) between Western and Eastern centers might influence surgical outcomes for pCCA patients, especially for BC type 4 patients.

Recently, Kimura et al.^32^ compared the long- and short-term outcomes for 183 patients who underwent radical resection for pCCA at an Eastern center (Hirosaki University Hospital, Japan) and a Western center (St. James's University Hospital, Leeds, UK). The authors reported a significant difference in the patient characteristics between the two centers. Moreover, although the 90-day mortality rates differed (2.5% vs 13.6%, respectively), the 5-year disease-specific survival rate was 32.8% for the Eastern center and 31.9% for the Western center (*p* = 0.77).^32^ Interestingly, in a preliminary analysis of the pCCA consortium data including three major hepatobiliary centers (Memorial Sloan Kettering Cancer Center, New York, NY; the Academic Medical Center in Amsterdam the Netherlands; Hokkaido University Graduate School of Medicine in Sapporo, Japan), the authors reported large overall differences in baseline characteristics, treatment, and clinical outcomes between an Eastern cohort (Japan) and a Western cohort (New York and Amsterdam) of patients undergoing combined hilar and liver resections for pCCA.^33^ Although the authors reported that the Western cohort had a worse survival and a higher 90-day mortality than the Eastern patients, after a PSM between the two cohorts, these differences were significantly attenuated.^33^

In our analysis, the 5-year OS was 30% for the BC types 2 and 3 pCCA patients and 23% for the BC type 4 pCCA patients. In a recent review by Ku et al.^17^ investigating the long-term outcomes for BC type 4 pCCA patients, the OS varied considerably among the different studies examining liver resection for BC type 4 pCCA, ranging for 10.5% at 3 years to 52% at 5 years.^10^

Ebata et al.^11^ investigated the oncologic outcomes of 216 patients who underwent liver resection for BC type 4 pCCA and reported a 5-year OS of 33%, ranging from 37% for the R0 patients (*n* = 154) to 53% for the N0 patients (*n* = 87). Percutaneous biliary drainage, blood transfusion, nodal status, and distant metastasis were predictors of poor patient prognosis.^11^

Similarly, in a multi-institutional analysis including 1352 pCCA patients who underwent surgical resection at eight Japanese centers, no differences in terms of 5-year OS among BC types 1 and 2, BC type 3, and BC type 4 N0/R0 patients were reported (5-year OS: BC types 1 and 2 [63%]; BC type 3 [66%]; BC type 4 [59%]; all *p* > 0.29). Li et al.^29^ analyzing the oncologic outcomes of 142 BC type 4 pCCA patients reported a 5-year OS of 25% for the R0 patients and 28% for the N0 patients.

The indication for resection in BC type 4 pCCA patients should be evaluated carefully in the context of the significant postoperative mortality and poor prognosis associated with negative prognostic factors (i.e., positive surgical margins, lymph-node metastasis). For these reasons, a careful selection of candidates for surgical resection and meticulous preoperative management are decisive for improvement of both the short- and long-term surgical outcomes.

In this article, we report the largest Western surgical series describing results of surgery for pCCA, emphasizing the potential benefit of surgery also for BC type 4 pCCA. Notably, promising results can be achieved for selected patients because in the sub-analysis for N0/R0 patients, the 5-year OS was 43% for the BC types 2 and 3 patients and 42% for the BC type 4 pCCA patients (*p* = 0.38).

The current study should be interpreted in light of several limitations. Although only tertiary referral centers were included in the collaborative group, given the retrospective and multi-institutional nature of the study, it was obviously not possible to standardize the surgical approaches and perioperative managements among the collaborating centers. During the study period, from 2000 to 2017, preoperative management and surgical treatments may have changed over time. Moreover, the indications for the surgical treatment of BC type 4 pCCA patients were based on single-center/surgeon preferences. Importantly, in the survival analysis, we included only patients who survived at least 30 days after surgery. Although 90-day mortality might be more significant, we believe that the occurrence of very early recurrence (i.e., before 3 months) might have influenced the results of our analysis. According to these considerations, the incidence of PHLF, biliary leak, Clavien-Dindo ≥3 complications, and 30-day mortality was greater among the BC type 4 patients than among the BC types 2 and 3 patients, but the difference was statistically nonsignificant. Conversely, the 90-day mortality was slightly higher among the BC types 2 and 3 patients (13.4%) than among the BC type 4 patients (12.2%), probably due to patients with a very early recurrence.

Unfortunately, due to the multicentric nature of our study, we were not able to collect the cause of death to compute the 90-day mortality for the survival analysis. Moreover, in the analysis, the impact of systemic treatment was not investigated. Although the use of adjuvant chemotherapy/radiotherapy was probably heterogeneous in the study population, further studies investigating the role of adjuvant treatments for BC type 4 pCCA patients should address this specific issue.

Finally, significant differences in terms of clinical-pathologic characteristics between the BC types 2 and 3 patients and the BC type 4 patients might have influenced the comparison of survival data. To mitigate the selection and confounding bias, an exact propensity score match was performed matching patients based on the most important preoperative clinicopathologic characteristics (i.e., age, sex, preoperative drainage, CA 19-9 level, preoperative PVE, and preoperative cholangitis).

In conclusion, surgery for the selected BC type 4 pCCA patients resulted in short- and long-term outcomes similar to those for the BC types 2 and 3 pCCA patients. Although the patients in the BC type 4 group underwent a more “aggressive” type of surgery (i.e., vascular resection, extended hepatectomies), the incidences of severe complications and mortality were comparable with those classified as BC types 2 and 3 pCCA. Likewise, although the BC type 4 patients had worse clinical-pathologic characteristics, these patients had oncologic outcomes similar to those of the BC types 2 and 3 pCCA patients. Surgery should therefore be considered an acceptable curative treatment option for selected patients with BC type 4 pCCA although it requires a more technically challenging approach.

## Supplementary Information

Below is the link to the electronic supplementary material.

Supplementary file1 (DOCX 28 kb)
