# Supplementary material for: Surgery for Bismuth-Corlette Type 4 Perihilar Cholangiocarcinoma: Results from a Western Multicenter Collaborative Group
Source: Ann Surg Oncol. 2021 May 6;28(12):7719–29. doi: 10.1245/s10434-021-09905-z (PMC8519825; doi:10.1245/s10434-021-09905-z)
Supplement: Supplementary file 1 — Supplementary file1 (DOCX 28 kb) [file 10434_2021_9905_MOESM1_ESM.docx]

**Figure S1** Overall survival of Bismuth type 2 or 3 N0R0 patients versus Bismuth type 4 N0R0 patients after propensity score-matching.

**TABLE S1** Baseline characteristics of perihilar cholangiocarcinoma patients with Bismuth type 4 (*n* = 308) versus Bismuth type 2 or 3 (*n* = 308) disease after propensity score-matching

| Variables | | | Bismuth type 4  *n* (%) | | | Bismuth type 2 or 3  *n* (%) | | | *p* Value | | | |  |
| --- | --- | --- | --- | --- | --- | --- | --- | --- | --- | --- | --- | --- | --- |
| No. of patients | | | 306 | | | 306 | | | — | | | |  |
| Age (years)  ≤65  >65 | | | 154 (50.3)  152 (49.7) | | | 154 (50.3)  152 (49.7) | | | >0.99 | | | |  |
| Gender  Male  Female | | | 175 (57.2)  131 (42.8) | | | 175 (57.2)  131 (42.8) | | | >0.99 | | | |  |
| ASA physical status classification  1–2  3–4 | 185 (62.3)  112 (37.7) | | | 176 (62.9)  104 (37.1) | | | | | >0.99 | | | |  |
| Preoperative biliary drainage  No  Yes | | | 26 (8.5)  280 (91.5) | | | 26 (8.4)  282 (91.6) | | | >0.99 | | | |  |
| Type of biliary drainage  No drainage  PTBD  Endoscopic biliary drainage  Both | | | 26 (8.5)  65 (21.2)  148 (48.4)  67 (21.9) | | | 26 (8.6)  113 (37.2)  130 (42.8)  35 (11.4) | | | <0.001 | | | |  |
| Preoperative cholangitis  No  Yes | | | 242 (79.1)  64 (20.9) | | | 242 (79.1)  64 (20.9) | | | >0.99 | | | |  |
| Portal vein embolization  Yes  No | | | 77 (25.2)  229 (74.8) | | | 77 (25.2)  229 (74.8) | | | >0.99 | | | |  |
| CA 19-9 (U/mL)  ≤100  >100 | | | 210 (68.6)  96 (31.4) | | | 210 (68.6)  96 (31.4) | | | >0.99 | | | |  |
| Major liver resection (≥3 segments)  Yes  No | | | 297 (97.1)  9 (2.9) | | | 306 (100.0)  - | | | 0.002 | | | |  |
| Extended liver resection (≥5 segments)  Yes  No | | | 185 (60.5)  121 (39.5) | | | 70 (22.9)  236 (73.7) | | | <0.001 | | | |  |
| Type of liver resection  Left hepatectomy  Extended left hepatectomy  Right hepatectomy  Extended right hepatectomy  Segments 4 and 5  Central Hepatectomy | | | 65 (21.2)  81 (26.5)  47 (15.4)  104 (34.0)  3 (1.0)  6 (1.9) | | | 214 (69.9)  33 (7.5)  22 (7.2)  47 (15.4)  —  — | | | <0.001 | | | |  |
| Caudate lobe resection  Yes  No  NA | | | 140 (45.8)  51 (16.7)  115 (37.5) | | | 197 (64.4)  50 (16.3)  59 (19.3) | | | <0.001 | | | |  |
| Portal vein resection  Yes  No | | | 151 (49.3)  155 (50.7) | | | 75 (24.5)  231 (75.5) | | | <0.001 | | | |  |
| Hepatic artery resection  Yes  No | | | 11 (3.6)  295 (96.4) | | | 12 (3.9)  294 (96.1) | | | 0.83 | | | |  |
| Perineural invasion  Yes  No  NA | | | 200 (65.4)  56 (18.3)  50 (16.3) | | | 204 (66.7)  71 (23.2)  31 (10.1) | | | 0.05 | | | |  |
| Tumor grade  Well/moderately  Poor/undifferentiated  NA | | | 218 (71.3)  69 (22.5)  19 (6.2) | | | 225 (73.6)  69 (22.5)  12 (3.9) | | | 0.43 | | | |  |
| Surgical margins  R0  R1 | | | 185 (60.5)  121 (39.5) | | | 208 (68.0)  98 (32.0) | | | 0.06 | | | |  |
| Lymphnodes status  N0  N1 | | | 166 (54.2)  140 (45.8) | | | 182 (59.5)  124 (40.5) | | | 0.19 | | | |  |
| Median hospital stay: days (IQR) | | | 16.0 days (10.0–26.0) | | | 12.0 days (11.0–13.0) | | | <0.001 | | | |  |
| Post-hepatectomy liver failure  No failure/PHLF A  PHLF B/C | 246 (80.4)  60 (19.6) | | | 269 (88.2)  36 (11.8) | | | | | 0.008 | | | |  |
| Biliary leak  No leak/grade A  Grade B/C | 235 (76.8)  71 (23.2) | | | 255 (83.9)  49 (16.1) | | | | | 0.027 | | | |  |
| Intra-abdominal abscess^a^  No  Yes | 285 (93.1)  21 (6.9) | | | 277 (90.5)  29 (9.5) | | | 0.24 | | | |  |  |  |
| Severe bleeding complications^a^  No  Yes | 295 (96.4)  11 (3.6) | | | 297 (97.1)  9 (2.9) | | | 0.65 | | | |  |  |  |
| Other severe complications^a^  No  Yes | 260 (85.0)  46 (15.0) | | | 262 (85.6)  44 (14.4) | | | 0.82 | | | |  |  |  |
| Clavien-Dindo ≥3 complications  No  Yes | | 149 (48.7)  157 (51.3) | | | 183 (60.0)  122 (40.0) | | | 0.005 | | | |  |  |
| No. of severe complications^a^  1  2  3  4 | 129 (82.2)  23 (14.6)  5 (3.2)  — | | | 95 (77.9)  21 (17.2)  4 (3.3)  2 (1.6) | | | 0.39 | | | |  |  |  |
| 30-Day mortality  No  Yes | | | 278 (90.8)  28 (9.2) | | | 288 (94.1)  18 (5.9) | | | 0.13 | | | |  |
| 90-Day mortality  No  Yes | | | 269 (87.9)  37 (12.1) | | | 271 (88.6)  35 (11.4) | | | 0.80 | | | |  |
| ASA, American Society of Anesthesiologists; PTBD, percutaneous transhepatic biliary drainage; NA, ; IQR, interquartile range; PHLF,  ^a^Clavien-Dindo ≥3 complications | | | | | | | | | |  |  |  |  |
|  | | | | | | | | | | | | | |

**TABLE S2.** Uni- and multivariable survival analysis: Cox’s proportional hazard model for overall survival after propensity score-matching

| Variables | Univariable | | Multivariable | |
| --- | --- | --- | --- | --- |
|  | HR (95 % CI) | *p* Value | HR (95 % CI) | *p* Value |
| Gender (male vs female) | 1.21 (0.91–1.62) | 0.18 |  |  |
| Age (≤65 vs >65 years) | 1.26 (0.95–1.66) | 0.11 |  |  |
| Bismuth type (2 or 3 vs 4) | 0.89 (0.67–1.1) | 0.42 |  |  |
| Biliary drainage (yes vs no) | 0.91 (0.53–1.54) | 0.73 |  |  |
| Portal vein embolization (yes vs no) | 1.29 (0.90–1.85) | 0.16 |  |  |
| Major liver resections (yes vs no) | 0.42 (0.05–3.05) | 0.39 |  |  |
| Extended liver resections (yes vs no) | 1.22 (0.91–1.64) | 0.18 |  |  |
| CA 19-9 (≤100 vs >100 U/mL) | 1.34 (0.98–1.82) | 0.04 |  |  |
| Segment 1 resected (yes vs no) | 0.88 (0.73–1.07) | 0.21 |  |  |
| Portal vein resection (yes vs no) | 1.10 (0.83–1.46) | 0.51 |  |  |
| Hepatic artery resection (yes vs no) | 1.64 (0.80–3.32) | 0.17 |  |  |
| Margins (positive vs negative) | 1.99 (1.48–2.67) | <0.001 | 1.70 (1.27–2.29) | <0.001 |
| N stage (N1 vs N0) | 2.13 (1.59–2.85) | <0.001 | 2.13 (1.58–2.85) | <0.001 |
| Perineural invasion (yes vs no) | 1.23 (0.93–1.62) | 0.14 |  |  |
| Differentiation grade (poor vs well/moderately) | 1.70 (1.24–2.33) | <0.001 | 1.28 (1.01–1.62) | 0.042 |

HR, hazard ratio; CI, confidence interval

**TABLE S3** Uni- and multivariable survival analysis: Cox’s proportional hazard model for disease-free survival after propensity score-matching

| Variables | Univariable | | Multivariable | |
| --- | --- | --- | --- | --- |
|  | HR (95 % CI) | *p* Value | HR (95 % CI) | *p* Value |
| Gender (male vs female) | 1.54 (1.16–2.04) | 0.002 | 1.46 (1.09–1.97) | 0.010 |
| Age (≤65 vs >65 years) | 0.95 (0.72–1.26) | 0.74 |  |  |
| Bismuth type (2 or 3 vs 4) | 1.13 (0.86–1.48) | 0.38 |  |  |
| Biliary drainage (yes vs no) | 0.53 (0.33–0.85) | 0.008 | 0.61 (0.38–1.01) | 0.051 |
| Portal vein embolization (yes vs no) | 0.85 (0.61–1.17) | 0.31 |  |  |
| Major liver resections (yes vs no) | 1.89 (0.47–7.63) | 0.37 |  |  |
| Extended liver resections (yes vs no) | 0.67 (0.51–0.89) | 0.005 |  |  |
| CA 19-9 (≤100 vs >100 U/mL) | 1.59 (1.19–2.13) | 0.002 | 1.40 (1.04–1.88) | 0.024 |
| Segment 1 resected (yes vs no) | 0.75 (0.62–0.91) | 0.003 | 0.76 (0.62–0.94) | 0.009 |
| Portal vein resection (yes vs no) | 0.73 (0.54–0.97) | 0.031 |  |  |
| Hepatic artery resection (yes vs no) | 1.69 (0.86–3.30) | 0.13 |  |  |
| Margins (positive vs negative) | 1.83 (1.38–2.41) | <0.001 | 1.65 (1.24–2.19) | <0.001 |
| N stage (N1 vs N0) | 2.06 (1.57–2.72) | <0.001 | 2.02 (1.53–2.69) | <0.001 |
| Perineural invasion (yes vs no) | 0.91 (0.73–1.14) | 0.42 |  |  |
| Differentiation grade (poor vs well/moderately) | 1.22 (0.98–1.53) | 0.07 | 1.21 (0.97–1.51) | 0.089 |

HR, hazard ratio; CI, confidence interval
